# Spatial Proteomics for the Molecular Characterization of Breast Cancer

**DOI:** 10.3390/proteomes11020017

**Published:** 2023-05-03

**Authors:** Klára Brožová, Brigitte Hantusch, Lukas Kenner, Klaus Kratochwill

**Affiliations:** 1 Core Facility Proteomics, Medical University of Vienna, 1090 Vienna, Austria; 2 Department of Pathology, Medical University of Vienna, 1090 Vienna, Austria; 3 Division of Molecular and Structural Preclinical Imaging, Department of Biomedical Imaging and Image-Guided Therapy, Medical University of Vienna, 1210 Vienna, Austria; 4 Unit of Laboratory Animal Pathology, University of Veterinary Medicine, 1090 Vienna, Austria; 5 CBmed GmbH—Center for Biomarker Research in Medicine, 8010 Graz, Austria; 6 Christian Doppler Laboratory for Applied Metabolomics, Medical University of Vienna, 1090 Vienna, Austria; 7 Christian Doppler Laboratory for Molecular Stress Research in Peritoneal Dialysis, Department of Pediatrics and Adolescent Medicine, Medical University of Vienna, 1090 Vienna, Austria; 8 Division of Pediatric Nephrology and Gastroenterology, Department of Pediatrics and Adolescent Medicine, Comprehensive Center for Pediatrics, Medical University of Vienna, 1090 Vienna, Austria

**Keywords:** breast cancer, proteomics, MALDI imaging, mass spectrometry imaging

## Abstract

Breast cancer (BC) is a major global health issue, affecting a significant proportion of the female population and contributing to high rates of mortality. One of the primary challenges in the treatment of BC is the disease’s heterogeneity, which can lead to ineffective therapies and poor patient outcomes. Spatial proteomics, which involves the study of protein localization within cells, offers a promising approach for understanding the biological processes that contribute to cellular heterogeneity within BC tissue. To fully leverage the potential of spatial proteomics, it is critical to identify early diagnostic biomarkers and therapeutic targets, and to understand protein expression levels and modifications. The subcellular localization of proteins is a key factor in their physiological function, making the study of subcellular localization a major challenge in cell biology. Achieving high resolution at the cellular and subcellular level is essential for obtaining an accurate spatial distribution of proteins, which in turn can enable the application of proteomics in clinical research. In this review, we present a comparison of current methods of spatial proteomics in BC, including untargeted and targeted strategies. Untargeted strategies enable the detection and analysis of proteins and peptides without a predetermined molecular focus, whereas targeted strategies allow the investigation of a predefined set of proteins or peptides of interest, overcoming the limitations associated with the stochastic nature of untargeted proteomics. By directly comparing these methods, we aim to provide insights into their strengths and limitations and their potential applications in BC research.

## 1. Introduction

Proteins fulfil different functions in different subcellular compartments, and their aberrant localization can lead to a wide range of diseases, including the development of cancer [1]. The ability to study how protein abundance and localization is altered in pathogenic cells can be useful for finding new biomarkers and developing new therapies [2]. Studying the spatial distribution of proteins within a cell reveals an additional level of regulation and function that cannot be inferred from gene and protein expression levels alone [3]. Spatial proteomics is an approach that allows the localization of proteins within cells and tissues to be studied, enabling understanding of the underlying biological processes, such as proliferation, growth and signaling pathways, that determine cellular heterogeneity within tissues [1]. This review will focus on the technique of imaging mass spectrometry, which can be used to analyze the distribution and abundance of proteins within a tissue or cell at a high resolution.

Oncology is a dynamic field that is developing dynamically both in terms of diagnostics and therapy. One of the main tasks of the life sciences, which include a wide variety of molecular biology, genomics and proteomics techniques, is to find biomarkers that can be used in diagnostics and in targeted oncological therapy. Undeniably, biomarkers for oncological diseases have fundamentally changed current diagnostic and therapeutic procedures [4] and can be classified as predictive or prognostic [5]. Breast cancer (BC) is the second most common cancer worldwide, with a mortality rate of still 25% (WHO data), making it one of the leading causes of death in the female population. This is mainly due to a lack of understanding of the heterogeneity of BC, which contributes to treatment failure and patient death [6]. BC is a highly heterogeneous disease in which both genetic and environmental factors play a critical role. Based on advanced analytical methods, a large number of BC-associated biomarkers involving DNA, RNA, proteins, and intact cells have been identified [7]. However, a deeper insight into the molecular and cellular pathways involved in BC pathogenesis may contribute to the development of improved therapeutic options [8].

## 2. Breast Cancer Diagnosis and Classification

The phenotypes of luminal and myoepithelial cells in the healthy mammary gland of the breast from which a tumor can develop are tightly controlled [9]. The diagnosis, prognosis and treatment options for BC are based on histological and phenotypic analysis of the tumors, which are currently graded based on tumor structure and cellular morphology. BC can be classified as carcinoma in situ or invasive carcinoma, carcinoma in situ can be further subdivided into ductal or lobular subtypes, and invasive BC is characterized by multiple histological findings [10]. The combination of immunofluorescence (IF) and fluorescence in situ hybridization (FISH) is routinely used for the clinical detection of cytogenetic abnormalities in BC [11]. The majority of BC expresses established clinical biomarkers, such as the estrogen receptor (ER), progesterone receptor (PR), human epidermal growth factor receptor 2 (HER2), and the proliferation marker Ki67 [12], which are used to guide treatment decisions and serve as surrogates for prognosis. These biomarkers categorize tumors as luminal A (ER+ and/or PR+, HER2−, Ki-67+ < 20%), luminal B (ER+ and/or PR+, HER2−, Ki-67+ ≥ 20%), luminal B-HER2+ (ER+ and/or PR+, HER2+), HER2+ (ER−PR−HER2+), and triple-negative (TN; ER−PR−HER2−) [13]. For an anti-receptor-based therapy decision, it is important to analyze whether more than 1% of BC cells express ER and PR hormone receptors and whether more than 10% of BC cells express higher levels of HER2 protein or show amplification of the HER2 gene [14,15]. 

To diagnose BC metastases, the primary tumor must be identified, using formalin-fixed paraffin-embedded (FFPE) tissue samples stained with hematoxylin and eosin (H&E) or immunohistochemistry (IHC) [16]. More recently, mutations have been detected using methods, such as PCR, in situ hybridization (ISH) [17], or exome gene sequencing, to classify metastases. The disadvantage of these techniques is that they mostly rely on a limited number of tumor characteristics and genetic variations [18].

Overall, the clinical BC classification does not adequately reflect tumor heterogeneity, and patients with the same diagnosis may have different outcomes, requiring better characterization of BC ecosystems. Treatment approaches should be based on the molecular characteristics of the specific tumor. For a detailed diagnosis of BC, the currently used prognostic biomarkers, which are mainly based on tumor characteristics (tumor grade, tumor size, lymph node metastases, and molecular features), are not sufficient [19]. Genomic and proteomic approaches can complement the information provided by routine determinations and, together with data analysis techniques, could significantly expand the information obtained [20]. 

BC subtypes differ at the immune cell level. Tumor-infiltrating lymphocytes and non-lymphocytic immune infiltrates, such as macrophages, can be present in BC [21,22]. This adds another layer of complexity to the development of personalized therapies. For example, tumor-associated macrophages that are recruited by the chemokine ligand 2 secreted by cancer cells promote the secretion of pro-angiogenic, pro-invasive and immunosuppressive factors [23,24], and represent promising therapeutic targets [25]. Understanding the spatial structure of the TME is very important for exploring the mechanisms that cause BC progression, invasion, metastasis, and angiogenesis and could help in designing new therapeutic approaches [26].

## 3. Molecular Biology of Breast Cancer

Significant differences in hormone receptor expression are major drivers of BC. In addition, alterations in various cell signaling pathways promote tumor cell proliferation, progression, and survival, such as the phosphatidylinositol 3-kinase/serine–threonine kinase (PI3K/AKT) the mitogen-activated protein kinase (MAPK) [8], the mammalian target of rapamycin (mTOR), the HER-2 tyrosine kinase [27], the Hedgehog [28], the tumor protein p53, and the phosphatase and tensin homolog (PTEN) [29] signaling pathways. For example, activation of the PI3K/Akt/mTOR pathway is a major cause of BC resistance to anti-tumor therapies. This makes the PI3K/Akt/mTOR signaling pathway a crucial object of study for understanding the development and progression of this disease [30]. As there are complicated interactions between these pathways, it is unclear how they interact with each other [31].

Therapeutic targets currently in use or are under development include the estrogen receptor (ER) and the androgen receptor (AR), growth factor receptors including HER2, the DNA-repair protein poly (ADP-ribose) polymerase (PARP), the apoptosis inhibitor proteins BCL-2 and survivin, the cell cycle proteins cyclin dependent kinases CDK4 and CDK6, the PI3K AKT and mTOR signaling proteins, and epigenetic enzymes such as methyltransferases (DNMTs) and histone deacetylases (HDACs) [32].

BC is characterized by altered metabolism, which leads to the differential expression of several metabolites and metabolic pathways, sustaining cell growth and proliferation. BC cells adaptively alter nutrient utilization during proliferation, a process known as metabolic transformation. The metabolic characteristics of different BC subtypes can provide insight into potential novel therapeutic targets [33]. A variety of factors, including extrinsic ones such as hypoxia, oxidative stress, and acidosis, and intrinsic factors, such as amplification of Myc proto-oncogene and mutations in the phosphatidylinositol-4,5-bisphosphate 3-kinase catalytic subunit (PIK3CA) and tumor protein p53 genes, can cause metabolic reprogramming in BC. Understanding the metabolic mechanisms involved in BC metastasis can provide important clues for the development of novel therapeutic approaches, particularly for the treatment of metastatic BC [34].

Another feature that increases BC heterogeneity is altered enzymatic, as well as non-enzymatic, post-translational protein modifications (PTMs). Deregulated PTM pathways may contribute to BC tumorigenesis and can be used as biomarkers. Some of these PTMs have been identified by mass spectrometry, antibody microarrays and immunohistochemical techniques [35]. Common PTMs in BC include phosphorylation [36], acetylation [37], and SUMOylation [38]. For example, SUMOylation is a PTM that plays an important role in many biological processes, such as cell cycle regulation, protein localization, transcription, and DNA damage repair, and deregulation of these pathways is observed in BC [39].

**Figure 1:**
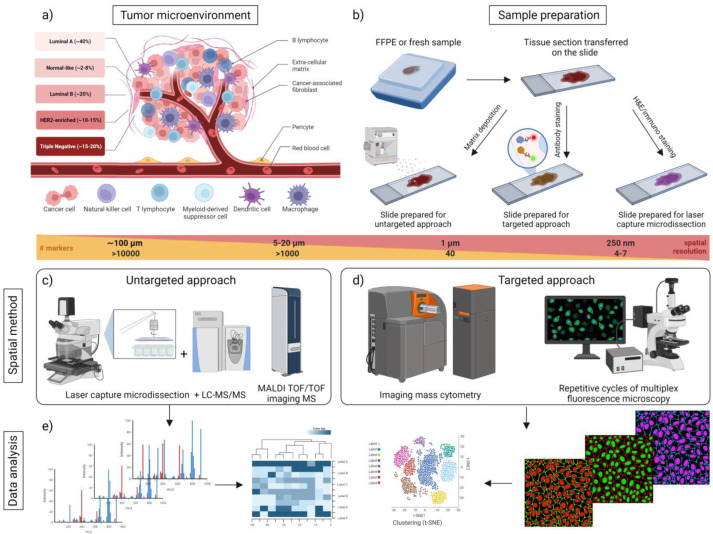
Overview of spatial proteomics approaches in breast cancer. (**a**) Tumor microenvironment in breast cancer. The tumor consists of tumor cells and stromal cells, including cancer-associated fibroblasts, mesenchymal cells, and immune cells, surrounded by an extracellular matrix. Interactions between these components within the tumor contribute to the optimal conditions for tumor cell proliferation, progression, and survival. To study these processes, a spatial approach is used to determine cellular heterogeneity within tissues. (**b**) Schematic outline of a typical workflow for fresh frozen (FF) or formalin-fixed paraffin-embedded (FFPE) tissue samples. Sample processing includes sectioning and mounting a tissue section on a target. (**c**) For untargeted methods, such as mass spectrometry imaging, a matrix solution is homogenously sprayed, the tissue surface is irradiated by the laser and ion images are generated. Small areas of tissue or cells isolated by laser capture microdissection (LCM) can be analyzed by highly sensitive liquid chromatography coupled to mass spectrometry (LC-MS). (**d**) For targeted methods, typically antibodies labeled with fluorophores or mass tags are applied for multiplex data acquisition. As the number of markers that can be analyzed increases, the spatial resolution substantially declines. (**e**) As all these methods produce data-rich outputs, computational analysis, such as dimension reduction, clustering, or network analysis, is applied to visually represent and calculate statistical information.

The tumor microenvironment (TME), consisting of the stroma, blood vessels and the immune system, also contributes to the heterogeneity of BC and is a valuable source of information for treatment options [40]. The tumor stroma contains fibroblasts, myofibroblasts, leukocytes, endothelial cells, macrophages, adipocytes and extracellular matrix (ECM) (Figure 1a). The stromal components constantly communicate and influence each other, allowing cancer cells to survive and develop [41]. For example, fibroblast-derived caveolin-1, which promotes breast cancer cell elongation, directional migration and metastasis, is expressed by cancer associated fibroblasts (CAFs) and is an important consequence of cancer cell–CAF interaction [42]. CAFs also express podoplanin, a transmembrane protein that contributes to the prognosis of invasive BC, indicating a highly aggressive BC subgroup. CAFs could be used as a selective target for anti-cancer therapies in invasive BC cases [43]. Overall, the TME induces the development of multiple cancer cell subpopulations that differ in their genetic, epigenetic and behavioral characteristics [44].

Each cell has unique proteomic, transcriptomic, and metabolomic features which creates functional differences. Single-cell approaches are providing new insights into cellular heterogeneity at the genomic, transcriptomic, proteomic, and metabolomic level [45]. Single-cell omics can be used to study complex biological processes, such as cell signaling [46], cell fate decisions [47], resistance to drugs [48], immune cell plasticity [49] and cellular dysregulation [50], that cannot easily be deciphered by studying individual genes, transcripts, and proteins [51].

Single cell omics, in contrast to bulk omics, targets extraction of DNA, RNA, proteins, and metabolites from individual cells within a tissue or cell culture to measure differences between cells and visualize discrete populations of cells based on their physiological condition. For detection and characterization of tumor heterogeneity, single-cell omics methods represent a promising approach [52].

## 4. Proteomics Technologies for Spatial BC Analysis

Omics technologies have transformed cancer research using both genomic, such as next-generation sequencing, and proteomic technologies [53]. To obtain information on the proteomic heterogeneity within tissues, analytical resolution at the cellular and subcellular level is crucial. The resulting accurate spatial distribution of proteins may allow the application of proteomics in clinical research [2] (Figure 1).

Proteins play a critical role in determining the functional status of cells in BC. As such, knowledge of protein expression levels and modifications is essential for understanding the heterogeneity of breast cancer and identifying early diagnostic biomarkers and therapeutic targets [54,55]. In addition, the subcellular localization of a protein is often closely related to its physiological function. However, characterizing the subcellular localization of proteins on a proteome-wide scale is challenging, particularly for translationally dynamic and multicompartmental proteins [56]. To address this challenge, a comprehensive understanding of the spatial structure of the TME assembly is necessary. The TME comprises a complex interplay between tumor cells and various stromal components, including cancer-associated fibroblasts, mesenchymal cells, and immune cells. Determining the spatial organization of these different cell types and their respective protein expression levels and modifications can provide insights into the mechanisms of tumorigenesis and the development of novel therapeutic strategies [26]. Moreover, such insights can help in the identification of specific protein–protein interactions and signaling pathways involved in breast cancer progression, enabling the development of targeted interventions that selectively modulate the activity of these pathways. Therefore, understanding the proteome and spatial organization of the TME is crucial for advancing our understanding of breast cancer biology and for developing effective treatment strategies. Furthermore, identification of proteomic differences in BC tissues before, during and after treatment could help with diagnosis, prognosis, and treatment selection. Proteomics, in combination with genomics, may improve the current BC classification and treatment decisions [57].

Numerous techniques have been developed to identify and characterize proteins at cellular and subcellular levels. Western blotting, which is based on immunoblotting, has long been used to study various aspects of proteins in disease diagnosis. Western blotting has the ability to detect different isoforms of proteins, protein–protein interactions, protein–DNA interactions, and post-translational modifications (PTMs), as well as their subcellular localization, typically in lysates of many cells or tissues [58]. Single-cell Western blotting on pore-gradient microgel arrays has been employed for the analysis of oncoprotein-related signaling by Duncombe et al. [59]. However, the validation of antibody specificities and the requirement for pre-subcellular fractionation remain significant weaknesses of this Western blotting application [58]. Although proteome-wide implementations of Western blotting do exist [60], two-dimensional gel electrophoresis methods are currently incompatible with single-cell or spatial proteome analysis. In contrast, the sensitivity of MS-based proteomics enables it to reach the single-cell level [61]. MS is a constantly evolving analytical technique for the detection and identification of metabolites, lipids, peptides, proteins, glycans, drugs, and metals [62]. The application of this technique for protein detection has led to the development of soft ionization techniques such as electrospray ionization (ESI) [63] and matrix-assisted laser desorption/ionization (MALDI) [64].

MS-based approaches can be either untargeted or targeted. The untargeted approach allows the analysis of proteins and peptides without a prespecified molecular focus, which is ideal for discovery research and unbiased hypothesis generation. In bulk proteomics, untargeted workflows often leverage the analytical power of extensive sample fractionation and long liquid chromatography (LC) gradients, both of which are challenging to apply to single-cell or spatial studies [65]. In contrast, targeted proteomics techniques are limited to a predefined set of proteins or peptides of interest. This pre-selection allows limitations associated with the stochastic nature of untargeted proteomics to be overcome. In addition to approaches using multiple reaction monitoring (MRM) or parallel reaction monitoring (PRM), targeted proteomic approaches often include the use of affinity tags and labels and face the challenges associated with the availability, quality, affinity, and specificity of antibodies [66]. These techniques include targeted MS, microscopy-based multiplex imaging and single-cell techniques, such as (imaging) mass cytometry using the CyTOF technology. A comparative account of the various technologies described in this review with their resolution abilities, advantages, and disadvantages in a tabular format is presented in Table 1.

### 4.1. Untargeted Spatial Proteomic Analysis (Untargeted MS and Imaging Mass Spectrometry)

Owing to the ability to perform high-throughput analyses, MS-based approaches have been at the center of attention for studying protein expression differences and localization [67]. An untargeted approach is often used in the initial phase of biomarker discovery, with the goal of maximizing the proteome coverage [68].

#### 4.1.1. Untargeted LC-MS for Spatial Proteomics

Untargeted proteomic approaches are further categorized according to whether intact proteins are analyzed directly or are first digested to peptides. The bottom-up approach involves proteolytic digestion, separation of peptides by liquid chromatography, generation of tandem mass spectra (MS/MS), and comparison of the peptide spectra with databases. In a bottom-up approach, information on the intact protein and its post-translational modifications are typically lost, making it difficult to correlate these data with MS data from intact proteins [69]. The top-down approach is performed without previous digestion of proteins and both intact and fragmented ion masses are measured, allowing full sequence coverage and characterization of proteoforms [70]. 

To obtain spatial information, tissue regions of interest can be isolated by microdissection techniques and subsequent protein extraction [71]. Recently, MS analysis has been successfully coupled with laser capture microdissection (LCM), which uses an ultraviolet laser to capture specific cells or regions of interest in a tissue section under the microscope [65,72] (Figure 1c). Isolation of tissue regions by LCM followed by protein extraction and analysis enables the spatial localization of proteins in individual cell populations within a heterogeneous tissue to be studied, to some extent. Mund et al. combined single-cell or single-nucleus laser microdissection with artificial-intelligence-driven image analysis of cellular phenotypes to quantify expressed proteins in a cell and identify targets for future drugs and diagnostics [65].

#### 4.1.2. Imaging Mass Spectrometry (IMS)

To gain insight into protein abundance in a tissue-specific context, MALDI IMS has been developed [73]. The workflow for peptide identification by MALDI IMS includes sample collection and storage, tissue sectioning, on-tissue protein digestion, matrix application, MALDI-TOF measurement and data analysis. Optimization of these parameters is crucial, as variances in tissue sampling and preparation affect MALDI IMS results. Both FFPE [74] and fresh frozen (FF) tissue samples can be used. FF samples must be frozen immediately after tissue sectioning and stored at −80 °C or below to prevent protein degradation and to preserve morphology [75]. The commonly used embedding using the “optimal cutting temperature” (OCT) medium is not recommended, as the OCT polymer interferes with the signals in MS analysis [76]. FFPE samples are very stable due to the formalin fixation, which makes them ideal for long-term storage without extensive cooling [77] but creates methylene bridges and methylol adducts that can be challenging for MS analysis [78,79]. Gelatin or carboxymethyl cellulose [80]-based embedding media have been shown to be more compatible with MALDI IMS. After embedding, the tissue is sectioned at 6–20 μm thickness at −20 °C and placed on electrically conductive slides, such as indium tin oxide (ITO) coated slides, which are commonly used and recommended for MALDI IMS [81] (Figure 1b).

In bottom-up approaches, protein cleavage is performed directly on the tissue section. This digestion step, most commonly using trypsin, can also release peptides from the chemically cross-linked FFPE tissue [82]. Another approach, by Angel et al., used collagenases and metalloproteinases to specifically target the spatial localization of collagen and elastin. These proteins make up the majority of the extracellular matrix and are tightly regulated during cancer progression, but their specific properties (insolubility, multiple PTMs) inhibit tryptic access [83].

After the on-tissue digestion step, the matrix is sprayed onto the specimen and co-crystallized with the sample. The matrix is an organic molecule that efficiently absorbs the wavelength of the light emitted by the laser and facilitates the peptide transfer into the gas phase [84]. It promotes ionization, acts as a solvent for the analyte, and helps to minimize aggregation of the analyte molecules [76]. α-cyano-4-hydroxycinnamic acid is commonly used for low molecular weight proteins, whereas sinapinic acid yields the best signals for high molecular weight proteins [76]. During MALDI ionization, mostly singly charged analytes are generated [85]. Other ionization techniques compatible with IMS, such as surface-enhanced laser desorption ionization (SELDI) desorption electrospray ionization (DESI) and secondary ion mass spectrometry (SIMS) have been developed [86]. However, to date, the most widely used ionization technique for protein and peptide identification in IMS is MALDI [87] (Figure 1c). The obtained ions in the gas-phase are then accelerated in an electric field and transferred towards the mass analyzer. In most cases, MALDI is coupled to a time-of-flight (TOF) analyzer. Ions of the same charge acquire the same kinetic energy; thus, lighter ions reach the detector faster than heavier ones [88]. 

The spatial resolution of IMS is 5–20 µm [89], and it has been shown that, in combination with other spatially resolved proteomics, it has the potential to resolve the heterogeneity of breast cancer metastasis and identify candidate drug targets specific to tumor cell clones to enable personalized treatments [90].

Identification of the analytes can be achieved by comparing the experimental m/z values of the analytes from the full MS scan with databases containing theoretical m/z values of known molecules digested with the same sequence-specific protease. A limitation of IMS is the practical impossibility to perform MS/MS measurement on the IMS instruments. There are two approaches to overcome this problem. Firstly, IMS data can be correlated with targeted (LC-MS/MS) data performed on another tissue to obtain complete structural information on protein analytes, including confident identification of analytes [91]. Casadonte et al. performed IMS to discriminate breast from pancreatic cancer metastases with an overall accuracy of 83.38%, a sensitivity of 85.95%, and a specificity of 76.96 % and identified peptides, such as heat shock protein beta-1, whose expression is associated with aggressive tumors and decreased survival in breast cancer and melanoma patients [18]. Heterogeneous nuclear ribonucleoprotein A2/B1 (hnRNPA2B1), a protein expressed in the cytoplasm and nucleus of breast cancer cells, has been identified as a novel prognostic biomarker candidate [92], and the cytoskeletal protein filamin A has been shown to potentially play a role in chemotherapy resistance in triple-negative breast cancer patients [93].

Secondly, to improve spatial resolution, sensitivity, and the ability to perform MS/MS identification of proteins has recently been established by the laser-induced post-ionization technique (also known as MALDI-2) in transmission-mode geometry (t-MALDI-2). A second laser beam initiates a secondary MALDI-like ionization processes in the gas phase by interacting with the analytes and the matrix evaporate generated by the first standard MALDI laser [94]. T-MALDI-2 uses a UV-laser-transmitting microscope objective, which is placed in close proximity to the back side of a sample, allowing it to achieve a resolution of less than 1 µm. Another advantage is that the ion optics and the laser optics are spatially separated, which permits optimization of both independently and therefore enables achievement of higher sensitivity [95]. The high sensitivity of t-MALDI-2 also allows the acquisition of MS/MS data at high resolution [96].

Despite the versatility of IMS mentioned above, there are still several limitations. For example, limited spatial resolution due to laser parameters, long data acquisition times, and ionic suppression in the low mass molecular analysis caused by the MALDI matrix and concomitants of the sample make it necessary to optimize the experimental parameters for each sample [97].

### 4.2. Targeted Spatial Proteomic Analysis

Targeted proteomic analysis refers to proteomic workflows that focus on a predefined list of proteins. Targeted methods include targeted mass spectrometry and antibody-based spatial proteomics.

#### 4.2.1. Targeted Mass Spectrometry

The assessment of already known candidate proteins using proteomic techniques is commonly used for the verification of biomarker candidates [98]. The main MS acquisition modes in targeted mass spectrometry are selected reaction monitoring (SRM)/multiple reaction monitoring (MRM) and parallel reaction monitoring (PRM) [99]. In SRM/MRM mode, specific peptides of selected m/z (parent or precursor ions) are isolated in the first mass analyzer quadrupole 1 (Q1) and subsequently fragmented in the second mass analyzer (Q2). These fragment ions (also called product ions) are monitored by a third mass analyzer (Q3) configured to filter ions of a specific m/z [100]. The PRM mode is based on an Orbitrap or TOF mass analyzer instead of the Q3 quadrupole and scans all product ions with high resolution and high accuracy [101]. Fragmentation in Q2 is typically achieved by collision-induced dissociation (CID), which works by collision of parent ions with a neutral gas [102]. Other commonly used fragmentation techniques are higher-energy collisional dissociation (HCD), surface-induced dissociation (SID) [103] and electron-transfer dissociation (ETD) [104].

Recently, Steiner et al. have used LC-MRM/MS for the targeted measurement of 200 proteins in FFPE samples of triple-negative, HER2-overexpressing and luminal A type breast tumors and obtained quantitative information for 185 of these proteins, including HER2, hormone receptors, Ki-67, and inflammation-related proteins, demonstrating that LC-MRM/MS can reliably measure proteins using surrogate peptides extracted from FFPE samples [105].

#### 4.2.2. Antibody-Based Spatial Proteomics (Imaging Techniques Using Single or Multiplexed Antibody Probes)

Although IHC is not a proteomic technique, it is the most commonly used technique that allows for the visualization of biomarkers in tissue, making it valuable for studying the morphological context and spatial distribution of these markers. In breast cancer diagnosis, clinicians routinely use a combination of IHC and FISH to detect ER, PR, and HER2, as well as expression of proliferation markers, such as Ki67 and programmed death ligand 1 (PD-L1), from core needle biopsies. This diagnostic approach has proved to be effective in providing accurate and timely information for clinical decision-making. Moreover, IHC can also be utilized to evaluate tumor-infiltrating lymphocytes, including their subtypes and spatial distribution, as well as to assess changes in genotype and phenotype between primary tumors and metastases [106]. However, despite its utility, IHC does have some limitations, such as the requirement for precise knowledge of the target antigen, the challenging nature of detecting post-translational modifications, and the restricted capacity of one marker per tissue section [107]. Additionally, the application of multiplex IHC, which allows for the simultaneous detection of multiple targets, is limited in clinical settings due to the spectral and spatial overlap among different markers [108]. IF is a type of immunohistochemistry technique that employs fluorophores to visualize a broad range of cellular antigens, including proteins [109]. The use of fluorescence microscopy enables spatial resolution ranging from single cells to single molecules [110]. Multiplex IF is a useful approach to the analysis of protein co-localization in cells, as well as in fresh or FFPE tissue samples [111]. One such method is cyclic IF, which involves repeated antibody staining and removal to evaluate biomarkers, as in the case of multiplexed fluorescence microscopy (Cell DIVE) [112] or tissue-based cyclic IF [113]. One limitation of IF techniques is that they often require repetitive cycles of primary antibody incubation, which can slow down the process of screening multiple protein markers [106]. However, the Cell DIVE multiplex imaging system is capable of performing over 30 rounds of staining and destaining, with up to three biomarkers identified at the end of each staining cycle, followed by computational processing. This allows for a more efficient and streamlined approach to analyzing multiple protein markers using IF techniques [114]. Another IF-based approach is co-detection by indexing (CODEX), a highly multiplexed spatial cytometry technique using DNA barcodes, fluorescent analogs of dNTP, and an in-situ polymerization-based indexing procedure. CODEX was first used for systemic characterization of splenic tissue structure in autoimmune disease [115] and has recently been applied by Mishra et al. in a breast cancer study [116].

IF can be more challenging for quantitative analysis due to issues with signal-to-noise ratios, background fluorescence, and tissue fixation. IHC can provide more consistent staining intensity and signal-to-noise ratios. A disadvantage of using a counterstain in IHC is potential non-specific background staining that can obscure the target stain. However, a counterstain can provide additional morphological information and improve the accuracy and specificity of the results. IF, typically, does not involve a counterstain, making it more challenging to identify specific cellular structures. The choice of counterstain and staining conditions must be carefully optimized for each specific experiment in both techniques [117]. 

Rojo et al. used multiplex IF to study the non-canonical NF-κB pathway, deregulated in breast tumors, and found that activation of this pathway was inversely associated with ER expression in ER positive breast cancer, predicting poor survival in this subgroup. They further assessed myoglobin expression as a possible surrogate marker for activation of the non-canonical NF-κB pathway in these tumors [118]. Kinkhabwala et al. recently introduced the MICS (MACSima Imaging Cyclic Staining) technology—IF imaging of hundreds of proteins at subcellular resolution. Multimarker analysis can identify potential targets for immunotherapy against solid tumors and is compatible with other imaging technologies [119].

Mass cytometry by time-of-flight (CyTOF) or mass cytometry imaging (MCI) utilizes laser ablation and inductively coupled plasma (LA-ICP) MS for detection of antibodies bound to single cells [120]. Cells or tissue sections immobilized on slides are therefore labeled with panels of antibodies against known structural or cell type-specific biomarkers which are conjugated to metal-chelating polymers carrying stable isotopes [121] (Figure 1b). After antibody incubation and washing, similar to IHC and IF techniques, the sample is ablated by a UV laser, generating a plume containing single cell information that is transferred to the ICP-MS via an argon stream. The metal ions derived from the conjugated antibodies are cooled into a focused beam of ions, which are then accelerated into a TOF analyzer [122]. The metal isotopes are measured simultaneously and linked to the location of each spot on the tissue. The tissue is scanned spot-by-spot along a scan line while the slide moves under the laser beam. Each metal isotope which is associated with a labeled antibody can be distinguished. The scan lines provide an intensity map of all target proteins within the tissue [121]. The “Hyperion” imaging system (Standard Biotools, formerly Fluidigm) can measure up to 40 parameters simultaneously in FFPE or FF tissue sections with subcellular resolution [123] (Figure 1d). The main advantage of this technique is that there is no sample autofluorescence, as often observed in IF, and no matrix artefacts as encountered in IMS [124].

Mass cytometry imaging has already been frequently applied in breast cancer research. For example, Ali et al. performed mass cytometry imaging to detect 37 protein markers in FFPE samples of 483 breast tumor specimens in combination with prior genomic characterization and revealed phenotypes of epithelial, stromal and immune cell types. Distinct combinations of cell phenotypes and cell–cell interactions were associated with genomic subtypes of breast cancer [125]. Wagner et al. used this technique to investigate the phenotypic diversity of tumor and immune cells in 144 human breast tumor samples, 46 non-tumor control samples, and 4 mammoplasty samples from breast cancer–free individuals. Increased amounts of programmed death ligand 1 (PD-L1) tumor-associated macrophages and exhausted T-cells were detected in high-grade ER-positive and ER-negative tumors [25].

Angelo et al. developed a multiplexed ion-beam imaging (MIBI) method using secondary ion mass spectrometry instead of ICP-MS in the CyTOF instrument to image metal-labelled antibodies. MIBI allows the simultaneous analysis of 40–100 targets with higher spatial resolution (200 nm). They compared this technique with mass cytometry imaging for analysis of human FFPE breast tumor sections stained with ten labels simultaneously. Both techniques showed comparable results and qualitative expression patterns [126].

Merritt et al. have developed digital spatial profiling (DSP), a non-destructive method for spatial profiling of protein and RNA using oligonucleotide detection technologies [127]. This technique has been applied by McCart Reed et al. to study whole sections of metaplastic BC on a tissue microarray. DSP uses unique oligonucleotides attached to antibodies with a photo-sensitive linker that is hybridized to the tissue section. The oligonucleotide tags are decoupled after exposure to UV light and deposited onto a plate for digital quantification using an nCounter system or an NGS readout. As it is a non-destructive method, the tissue can be reused for other imaging techniques. This method allows selection of regions of interest with fluorescently labeled antibodies, for example, CD45 for lymphocytes and pan-cytokeratin for epithelial cells [128].

As mentioned above, IMS in principle is an untargeted method, however, Yagnik et al. have developed targeted IMS in combination with IHC (called MALDI-IHC), a matrix-free laser desorption/ionization method using photocleavable modified polypeptides that are subsequently cleaved and ionized during MSI. The spatial resolution of 10 μm allows mapping the spatial distribution and colocalization in BC samples [129].

## 5. Concluding Remarks and Outlook

Improving the diagnosis and treatment of breast cancer (BC) requires a comprehensive understanding of the TME in its tissue context. TME plays a crucial role in all stages of BC development, from early-stage progression to the aggressive growth and metastasis of tumors. Multiple cell types interact in a highly coordinated manner to facilitate the delivery of nutrients and oxygen to individual cancer cells and to evade natural checkpoints for uncontrolled growth. These interactions between cancer cells and the tumor microenvironment are mediated by various signaling pathways and can contribute to the development of drug resistance, making it critical to study the tumor microenvironment in order to identify new therapeutic targets and improve patient outcomes.

Proteomic methods offer a holistic approach that is attractive for identifying novel biomarkers and therapeutic targets. In the emerging field of spatial proteomics, the goal is to perform location-resolved proteome analysis while maintaining as much proteome coverage as possible. Untargeted proteomic approaches are more suitable for unbiased identification of unknown players, whereas targeted approaches are typically used for assessing and validating existing biomarker candidates. These approaches can be used to gain a better understanding of the complex interplay between multiple cell types in the tumor microenvironment during all stages of breast cancer development. By identifying novel biomarkers and therapeutic targets, proteomic methods may lead to improved diagnosis and treatment options for breast cancer patients.

Proteomic techniques such as liquid chromatography-mass spectrometry (LC-MS) and matrix-assisted laser desorption/ionization mass spectrometry (MALDI-MS) are data-rich methods that present significant analytical challenges when applied to two-dimensional or three-dimensional imaging. Despite these challenges, the comprehensive information provided by these techniques makes them attractive for the identification of novel biomarkers and drug targets in the field of spatial proteomics. MALDI-imaging datasets, for instance, can consist of thousands of spectra that represent hundreds of distinct molecules [130]. These large datasets require sophisticated computational methods for data reduction, image construction, and statistical analysis. To analyze the massive amounts of raw data generated by data-heavy proteomic techniques, a variety of bioinformatic operations must be performed, including normalization, baseline correction, spectra recalibration, smoothing, and data compression [131]. Data reduction methods are necessary to handle the high dimensionality of the dataset and may involve techniques such as principal component analysis (PCA) and related analyses to convert potentially correlated variables into a set of linearly uncorrelated values. Spatial segmentation, for example through clustering methods, can then be used to visualize potential patterns in the data [132]. However, with the increasing computational power available in proteomics, particularly with the application of machine learning (ML) and artificial intelligence (AI)-based methods, this field is expected to undergo significant transformation in the coming years [65,133] (Figure 1e).

The use of proteomic and spatial proteomic methods has become increasingly prevalent in breast cancer research in recent years, as evidenced in this review. The combination of untargeted MALDI imaging mass spectrometry (IMS) with targeted imaging mass cytometry (IMC) shows promise in identifying diagnostic and prognostic biomarkers and understanding tumor heterogeneity. It is imperative to further develop and enhance spatial proteomic techniques for faster and more accurate cancer research and personalized medicine.

## Figures and Tables

**Table 1:** Key features of spatial technologies for protein profiling in breast cancer tissue.

Type	Spatial Method	Principle	Spatial Resolution	Multiplexing	Advantage	Disadvantage
Targeted	IF	Antibodies designed to target specific proteins	250 nm	1–5	Signal amplification Resolution Analytical capabilities	Background signal and spectral overlap
	Cell DIVE	Antibodies with cyclic oligo-barcoded reporter	1 µm	>60	Standardized workflows with automation	Restricted to regions of interestPotential for epitope loss
	CODEX	Antibodies with cyclic oligo-barcoded reporter	1 µm	>60	Standardized workflows with automation	Restricted to regions of interest
	MCI	Combination of metal-labeled antibody immunostaining and ultraviolet laser ablation	1 µm	40	Minimal overlap or signal background	Requirement for expensive instrumentation and metal isotope-labeled antibodies
	MIBI	Combination of metal-labeled antibody immunostaining and ion-beam gun ablation	1 µm	40–100	Minimal overlap or signalbackground	Requirement for expensive instrumentation and metal isotope-labeled antibodies
	MICS	Photobleaching of fluorescent labels of recombinant antibodies and release of antibodies or their labels	1 µm	>100	Compatible with other technologies	Duration of experiment
	DSP	UV-cleaved oligo-conjugated primary antibody and barcode counting	5 µm	90	High multiplexing ability Non-destructive procedure	Restricted to regions of interest
	MALDI-IHC	Targeted IMS in combination with IHC	5–10 µm	12	Nondestructive method No cyclic workflows required	Extra preparation steps Limited sensitivity High acquisition time
Untargeted	t-MALDI-2	Laser-induced post-ionization technique in transmission-mode geometry	<1 µm	>100	Label-free conditions Compatible with subsequent H&E staining	Extra preparation steps Vacuum condition High acquisition time
	MALDI-IMS	Ionization of all molecules within the pixel, generating a separate spectra per pixel	5–20 µm	>1000	Label-free conditions Compatible with subsequent H&E staining	Extra preparation steps Vacuum conditionLimit of detection High acquisition time
	LCM + LC-MS/MS	Isolation of specific cells on a tissue section using laser	100 µm	>10,000	Label-free conditions Ability to isolate specific cell types from heterogeneous tissues	Extra preparation steps Need for a pathologist

Legend: IF: immunofluorescence, CODEX: co-detection by indexing, Cell DIVE: multiplexed fluorescence microscopy, MCI: mass cytometry imaging, MIBI: multiplexed ion-beam imaging, MICS: MACSima imaging cyclic staining, DSP: digital spatial profiling, MALDI-IMS: matrix-assisted laser desorption/ionization imaging mass spectrometry, IHC: immunohistochemistry, LCM: laser capture microdissection, LC-MS/MS: liquid chromatography–tandem mass spectrometry, t-MALDI-2: transmission-mode MALDI with laser post-ionization, H&E: hematoxylin and eosin.

## Data Availability

Not applicable.
